# Did aetiology matter in illness duration and complications in patients presenting in primary care with acute respiratory tract infections early in the COVID-19 pandemic: An observational study in nine countries

**DOI:** 10.1080/13814788.2024.2376084

**Published:** 2024-07-12

**Authors:** Roderick P. Venekamp, Marinus J.C. Eijkemans, Nicolaas P.A. Zuithoff, Femke Böhmer, Slawomir Chlabicz, Annelies Colliers, Ana García-Sangenís, Lile Malania, Jozsef Pauer, Angela Tomacinschii, Theo J. Verheij, Herman Goossens, Akke Vellinga, Christopher C. Butler, Alike W. van der Velden

**Affiliations:** a Julius Center for Health Sciences and Primary Care, University Medical Center Utrecht, Utrecht University, Utrecht, the Netherlands; b Institute of General Practice, Rostock University Medical Center, Rostock, Germany; c Department of Family Medicine, Medical University of Bialystok, Poland; d Department of Family Medicine & Population Health, University of Antwerp, Antwerp, Belgium; e Institut Universitari d‘Investigació en Atenció Primària Jordi Gol (IDIAP Jordi Gol), Barcelona, Spain; f National Center for Disease Control and Public Health, Tbilisi and Arner Science Management LLC, Tbilisi, GA, USA; g DRC Drug Research Centre, Balatonfüred, Hungary; h University Clinic of Primary Medical Assistance of State University of Medicine and Pharmacy “N. Testemițanu”, Chişinǎu, the Republic of Moldova; i Laboratory of Medical Microbiology, Vaccine & Infectious Disease Institute, University of Antwerp, Antwerp, Belgium; j School of Public Health, Physiotherapy and Sports Science, University College Dublin (UCD), Dublin, Ireland; k Nuffield Department of Primary Care Health Sciences, University of Oxford, Oxford, UK

**Keywords:** SARS-CoV-2, covid-19, respiratory tract infection, primary care, prediction

## Abstract

**Background:**

Despite considerable research into COVID-19 sequelae, little is known about differences in illness duration and complications in patients presenting in primary care with symptoms of acute respiratory tract infections (RTI) that are and are not attributed to SARS-CoV-2 infection.

**Objective:**

To explore whether aetiology impacted course of illness and prediction of complications in patients presenting in primary care with symptoms of RTI early in the COVID-19 pandemic.

**Methods:**

Between April 2020-March 2021 general practitioners from nine European countries recruited consecutively contacting patients with RTI symptoms. At baseline, an oropharyngeal-nasal swab was obtained for aetiology determination using PCR after follow-up of 28 days. Time to self-reported recovery was analysed with Kaplan-Meier curves. Predictors (baseline variables of demographics, patient and disease characteristics) of a complicated course (composite of hospital admission and persisting signs/symptoms at 28 days follow-up) were explored with logistic regression modelling.

**Results:**

Of 855 patients with RTI symptoms, 237 (27.7%) tested SARS-CoV-2 positive. The proportion not feeling fully recovered (15.6% vs 18.1%, *p* = 0.39), reporting being extremely tired (9.7% vs 12.8%, *p* = 0.21), and not having returned to usual daily activities (18.1% vs 14.4%, *p* = 0.18) at day 28 were comparable between SARS-CoV-2 positive (*n* = 237) and negative (*n* = 618) groups. However, among those feeling fully recovered (SARS-CoV-2 positive: 200 patients, SARS-CoV-2 negative: 506 patients), time to full recovery was significantly longer in SARS-CoV-2 patients (10.6 vs 7.7 days, *p* < 0.001). We found no evidence that predictors of a complicated course differed between groups (*p* = 0.07).

**Conclusion:**

Early in the pandemic, the proportion of patients not feeling fully recovered by 28 days was similar between SARS-CoV-2 positive and negative patients presenting in primary care with RTI symptoms, but it took somewhat longer for SARS-CoV-2 patients to feel fully recovered. More research is needed on predictors of a complicated course in RTI.

## KEY MESSAGES

Our primary care-based observational study found that recovery by 28 days was comparable between SARS-CoV-2 positive and negative RTI patients.

Future research is needed to unravel which host- and pathogen-related profiles are associated with higher risk of complications and persisting symptoms among patients presenting in primary care with RTI symptoms.

## Introduction

Initial reports of symptom duration and outcomes in COVID-19 patients were from hospitalised patients but with the evolving pandemic, COVID-19 sequalae were increasingly described in outpatients [1–6]. These studies indicate that 28-64% of patients reported persisting symptoms for more than one month, and up to a year. In contextualising these estimates, so called long-COVID received considerable media attention which might have influenced patients’ symptom perception and reporting. Moreover, these reports lacked an adequate comparison group. This is an important omission because post-infectious syndromes have been reported in other infectious diseases as well [7,8]. The need for further studies aimed at mitigating the risk of bias due to subjective outcome reporting has been underlined by a cross-sectional analysis of a population-based cohort study of 26,823 individuals in France. In this study, conducted between December 2020 and January 2021, patients with self-reported COVID-19 reported persisting physical symptoms at 10-12 months more frequently than those without self-reported COVID-19 [9]. The differences, however, were no longer apparent, except for anosmia, when comparing SARS-CoV-2 serology positive with negative participants. Whether such findings also apply to the broad population of patients presenting with RTI in primary care remains to be elucidated. In addition, it is currently unknown whether potential predictors of a complicated course of illness, persisting symptoms and/or hospitalisation, differ between those with and without confirmed SARS-CoV-2 infection.

We conducted a prospective observational cohort study in nine European countries early in the COVID-19 pandemic, when large-scale routine testing was not implemented yet, to explore whether aetiology impacted course of illness and prediction of complications in patients presenting in primary care with RTI symptoms.

## Methods

The SARS-CoV-2 Observational Study was implemented in nine European countries (Belgium, Germany, Spain, Georgia, Hungary, Ireland, Moldova, the Netherlands and Poland) between 14 April 2020 to 26 March 2021. The study protocol was approved by Ethics Committees in each participating country.

### Design, study population and data collection

The rationale, design and set-up of the study were described elsewhere [10] and a more extensive description of the methodology can be found in the Supplementary File. In short, general practitioners (GPs) recruited consecutively contacting patients aged one year or older presenting with RTI symptoms less than 14 days of unknown aetiology. At baseline, the GP completed a short questionnaire including information about patients’ sex, age, BMI status, smoking status, comorbidities, overall illness severity based on GP’s clinical evaluation of the patient without further guidance provided, signs and symptoms, and measurement of clinical parameters including body temperature, peripheral oxygen saturation, heart rate and respiratory rate. Next, a combined oropharyngeal and nasal swab (both nostrils) was obtained from all patients and transported to a central laboratory in Antwerp for issues of rigour. After completion of patient’s follow-up, samples were analysed for viral and bacterial pathogens using PCR. Details about all PCR results of participants have been described elsewhere [10].

At 7 and 28 days after inclusion, patients were contacted by phone. Patients were first asked whether they did feel fully recovered from their illness and if so at what day. Next, the same question was asked for individual symptoms (including shortness of breath and extreme tiredness), and return to usual daily activities). Finally, patients were questioned about any hospital admission (with/without overnight stay) during follow-up.

### Outcomes

The primary outcome was time to feeling fully recovered. Further exploratory outcomes were time to i) return to usual daily activities, ii) resolution of shortness of breath and iii) resolution of extreme tiredness. A complicated course of illness was defined when a patient was 1) admitted to hospital with overnight stay within 28 days, and/or 2) not yet feeling fully recovered at day 28, and/or 3) still experiencing shortness of breath and/or extreme tiredness and/or not having returned to usual daily activities at day 28.

### Statistical analyses

Baseline characteristics were tabulated. Differences between the SARS-CoV-2 positive and negative groups were determined using the Chi-square test for dichotomous variables and Student’s t test for continuous variables.

To determine time to full recovery in patients with and without PCR-confirmed SARS-CoV-2 infection, crude and inverse probability weighting (IPW) adjusted Kaplan-Meier curves were plotted taking age, sex, co-morbidity, BMI status, smoking, and overall illness severity into account. Data were censored on day 28. For time to resolution of shortness of breath, extreme tiredness, and return to usual daily activities, crude Kaplan-Meier curves were plotted. By default, log-rank tests were used to test for differences between groups. We evaluated the proportional hazard assumption visually, and if violated in case of non-proportionality, the Peto&Peto modification of the Gehan-Wilcoxon test was used.

To explore whether predictors of a complicated course differed between patients with and without PCR-confirmed SARS-CoV-2 infection, we included all candidate predictors (age, sex, co-morbidity, BMI category, smoking, and overall illness severity) and SARS-CoV-2 status as main effects, together with SARS-CoV-2*predictor interaction terms for all predictors in a logistic regression model. The pooled likelihood ratio test for interactions was used to assess between-group difference (p-value of <0.05).

All analyses were performed using R version 3.6.3 (R Foundation for Statistical Computing, Vienna, Austria).

## Results

Of the 876 included patients, 855 (97.6%) had both PCR-analysis results and complete follow-up data available and were included in the analysis. The 21 excluded patients were more often male, on average younger, and with less comorbidity, as compared to the 855 included ones.

Included patients’ mean age was 39 years and 55% (474/855) were female (Table 1). The majority had normal weight, a quarter had at least one comorbidity and overall illness severity was judged mild (583/855, 69%) or moderate (259/855, 31%) by the GP (Table 1).

**Table 1.** Baseline characteristics of 855 patients with acute RTI symptoms included in a primary care-based prospective observational study in nine European countries (April 2020-March 2021).

	Total (*n* = 855)	SARS-CoV-2 positive (*n* = 237)	SARS-CoV-2 negative (*n* = 618)	p-value ^
Female sex, n (%)	474 (55.4)	119 (50.2)	355 (57.4)	0.06
Mean age in years (SD)	38.8 (16.0)	40.9 (14.1)	38.1 (16.6)	0.02
BMI in mg/kg category, n (%)*				0.41
*Underweight (<18.5)*	11 (1.4)	1 (0.5)	10 (1.8)	
*Normal weight (18.5 to <25)*	542 (71.2)	149 (70.0)	393 (71.2)	
*Overweight (25 to <30)*	208 (27.3%)	63 (29.6)	145 (26.5)	
*Obesity (≥30)*	0 (0)	0 (0)	0 (0)	
Smoking status, n (%)*				0.12
*Current*	177 (21.3)	39 (16.7)	138 (23.1)	
*Previously*	79 (9.5)	25 (10.7)	54 (9.0)	
*Never*	575 (69.2)	170 (72.6)	405 (67.8)	
Any comorbidity^∼^, n (%)	238 (27.8)	68 (28.7)	170 (27.5)	0.73
Chronic lung disease, n (%)	71 (8.3)	17 (7.2)	54 (8.7)	0.46
Mean body temperature, Celsius (SD) [range]*	37.0 (0.9) [34.0-39.9]	37.3 (0.9) [35.1-39.8]	36.9 (0.9) [34.0-39.9]	<0.001
Mean peripheral oxygen saturation (SD) [range]*	96.7 (8.0) [73-100]	96.8 (8.2) [77-100]	96.7 (7.9) [73-100]	0.99
Mean heart rate (SD) [range]*	84 (14) [47-145]	84 (13) [47-145]	84 (14) [50-130]	0.87
Mean respiratory rate (SD) [range]*	17 (6) [11-79]	18 (6) [12-79]	17 (7) [11-78]	0.08
Overall illness severity based on GP’s judgement				0.09
*Mild*	583 (69.2)	153 (65.1)	430 (70.7)	
*Moderate*	259 (30.7)	81 (34.5)	178 (29.3)	
*Severe*	1 (0.1)	1 (0.4)	0 (0)	
Shortness of breath, n (%)	146 (17.1)	41 (17.3)	105 (17.0)	0.97
Extreme tiredness, n (%)	457 (53.5)	153 (64.6)	304 (49.2)	<0.001

BMI = body mass index; GP = general practitioner; SD: standard deviation.

^^^p-value for difference in baseline characteristics between SARS-CoV-2 positive and negative group.

*Missing total (and stratified to SARS-CoV-2 positive and negative group) for BMI: n = 94 (n = 24 and n = 70); Smoking status: *n* = 24 (*n* = 3 and *n* = 21); Body temperature: *n* = 151 (*n* = 36 and *n* = 115); Peripheral oxygen saturation: *n* = 340 (*n* = 198 and *n* = 232); Heart rate: *n* = 280 (*n* = 78 and *n* = 202); Respiratory rate: *n* = 376 (*n* = 98 and *n* = 278); Overall illness severity: *n* = 12 (*n* = 2 and *n* = 10).

^∼^*Any comorbidity: composite of chronic respiratory condition (asthma, COPD, cystic fibrosis), diabetes, cardiovascular disease, neoplasm, chronic kidney failure, chronic* neurological condition, immunocompromised, other.

Of the 855 included patients, 237 (27.7%) tested positive for SARS-CoV-2, 208 (24.3%) tested positive for other viruses, 311 (36.4%) tested positive for a bacterium, whereas in 99 patients (11.6%) no pathogen was detected.

No statistically significant difference in baseline characteristics were observed between patients with and without PCR-confirmed SARS-CoV-2 infection, except for age and body temperature; patients who tested SARS-CoV-2 positive were slightly older (mean age 41 versus 38 years) and had slightly higher body temperature (mean 37.3 versus 36.9 degrees Celcius) (Table 1). Peripheral oxygen saturation levels did not differ significantly between SARS-CoV-2 positive and negative patients (Table 1). However, of patients with a peripheral oxygen saturation value below 94% (*n* = 18), 17 tested negative for SARS-CoV-2.

### Course of illness: Comparison between SARS-CoV-2 positive and negative patients

At day 28, the proportions of patients not fully recovered (15.6% versus 18.1%, *p* = 0.39), and not returned to their usual daily activities (18.1% versus 14.4%, *p* = 0.18) at day 28 were comparable between the SARS-CoV-2 positive (*n* = 273) and negative group (*n* = 618). Similarly, the proportion of patients reporting being extremely tired at day 28 (9.7% versus 12.8%, *p* = 0.21) did not differ significantly. However, less patients in the SARS-CoV-2 positive group reported shortness of breath than in the SARS-CoV-2 negative group (4.6% versus 9.5%, *p* = 0.02).

**Figure 1.**
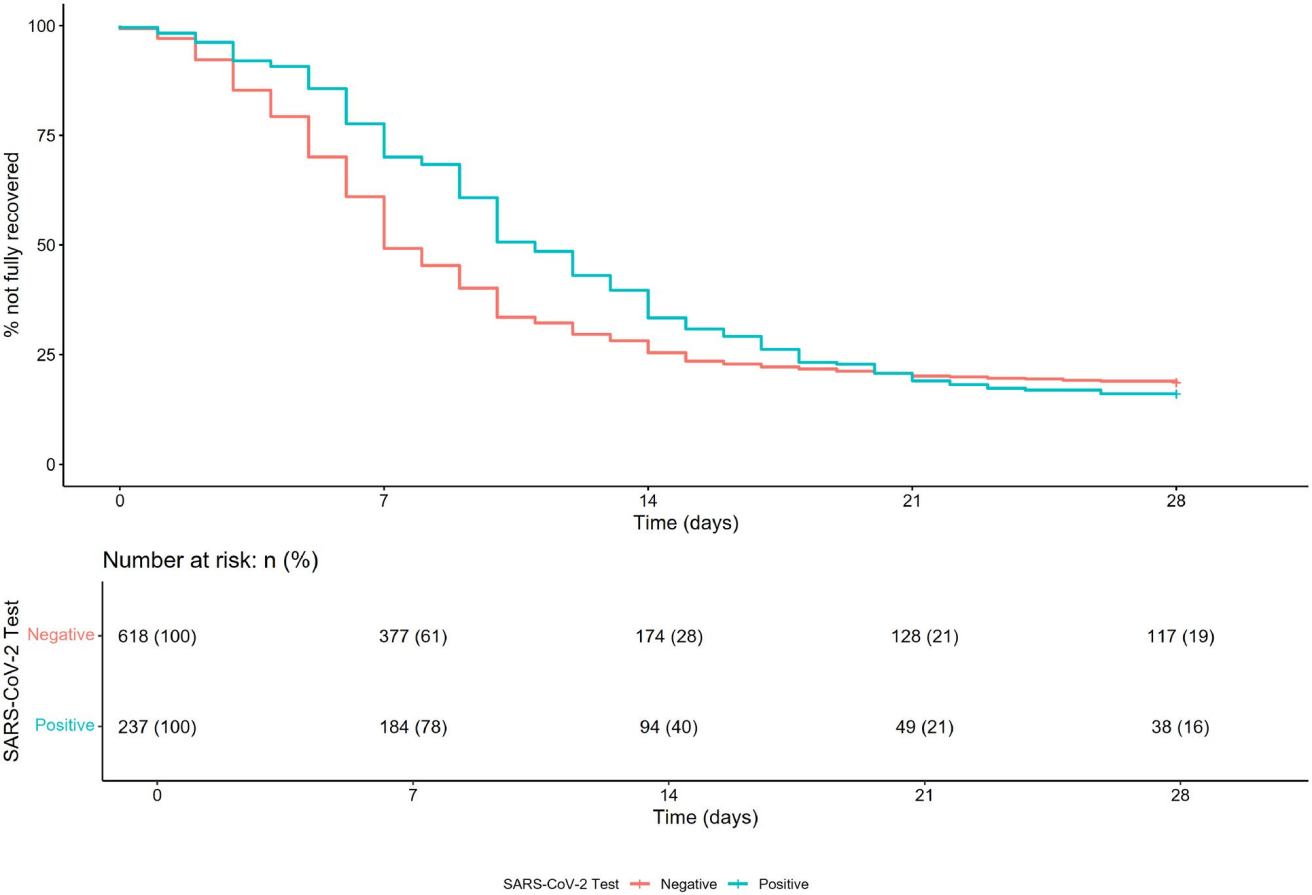
Crude Kaplan-Meier curves for time to reported feeling fully recovered in patients with acute RTI symptoms included in a primary care-based prospective observational study in nine European countries (April 2020-March 2021) with and without PCR-confirmed SARS-CoV-2 infection.

**Figure 2.**
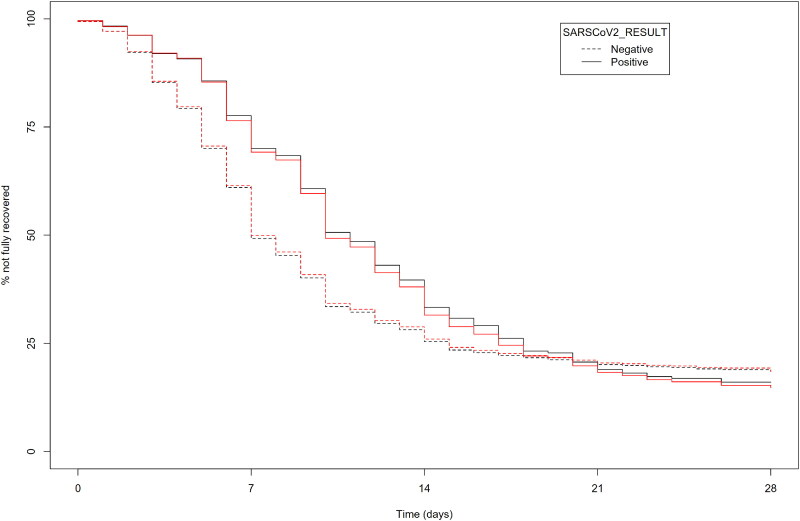
Inverse probability weighting adjusted Kaplan-Meier curves for reported feeling fully recovered in patients with acute RTI symptoms included in a primary care-based prospective observational study in nine European countries (April 2020-March 2021) with and without PCR-confirmed SARS-CoV-2 infection. Legend Figure 2: Age, sex, co-morbidity, BMI status, smoking, and overall illness severity were taken into account in the analysis. Red lines indicate the inverse probability weighting adjusted curves. See Figure 1 for absolute (i.e. unweighted) numbers at risk.

The crude and IPW-adjusted Kaplan-Meier curves for time to full recovery in the SARS-CoV-2 positive and negative groups are presented in Figures 1 and 2. These figures illustrate that time to full recovery was significantly longer in the SARS-CoV-2 positive group (*p* < 0.001, Peto&Peto modification of the Gehan-Wilcoxon test). Kaplan-Meier curves for time to resolution of shortness of breath, extreme tiredness and return to usual daily activities are presented in Supplementary Figures 1-3. There was no significant difference between the SARS-CoV-2 positive and negative groups in time to resolution of shortness of breath (*p* = 0.08, log-rank test).

The mean number of days (SD) to full recovery, return to their usual daily activities and not being extremely tired, was significantly longer in the SARS-CoV-2 positive group (10.6 (5.8) versus 7.7 (5.2) days, 11.5 (6.0) versus 7.4 (4.7) days, and 10.2 (5.6) versus 7.0 (4.7) days, respectively; *p* < 0.001 for all comparisons).

### Complicated course of illness

Of the 855 patients, 31 (3.6%) were admitted to hospital: 25 (10.5%) SARS-CoV-2 positive versus 6 (1.0%) SARS-CoV-2 negative patients. Due to the higher hospital admissions in the SARS-CoV-2 positive group (*n* = 273) than in the SARS-CoV-2 negative group (*n* = 618), the overall proportion of patients with a pre-defined complicated course of illness was slightly higher in this group: 31.2% versus 24.4% (*p* = 0.04). We found no evidence that candidate predictors of a complicated course (age, sex, co-morbidity, BMI ­category, smoking, and overall illness severity) ­differed between SARS-CoV-2 positive and negative groups (*p* = 0.07, pooled likelihood ratio test for interactions).

## Discussion

### Main findings

Our Europe-wide prospective cohort study including 855 patients presenting to primary care with RTI early in the COVID-19 pandemic showed that the proportions reported being fully recovered by 28 days were similar for patients who had a PCR-confirmed SARS-CoV-2 infection and those who were negative for SARS-CoV-2. However, full recovery within the 28-days follow-up period was on average about 3 days longer for SARS-CoV-2 positive patients. In addition, we found no evidence that candidate predictors of a complicated course (age, sex, co-morbidity, BMI category, smoking, and overall illness severity) differed between the SARS-CoV-2 positive and negative patient groups.

### Strengths and limitations

The study was conducted early in the COVID-19 pandemic when large-scale routine testing was not implemented yet and PCR-analysis was performed after completion of patient’s follow-up which allowed us to mitigate the risk of bias due to subjective outcome reporting. Furthermore, the prospective cohort design allowed for inclusion of both an unselected group of patients presenting to primary care with RTI symptoms irrespective of disease severity and an appropriate comparison group of patients with RTI symptoms but no PCR-confirmed SARS-CoV-2 infection.

Our study also has some limitations. First, our study was performed when the alpha variant was dominant, with unvaccinated patients. With vaccination and improved immunity a more favourable course of illness and lower risk of complications can be expected in SARS-CoV-2 positive patients. A similar study conducted in a more recent period would most likely show even smaller differences between SARS-CoV-2 positive and negative patients, with respect to time to recovery and hospital admission. Second, since follow-up was for 28 days any relevant longer-term differences in recovery and/or impact could not have been assessed. Given the attention for long-COVID, progressing after 28 days, follow-up of up to one year, could have added more insight into true incidence of long-COVID, whether other viral aetiologies also result in long-term consequences and risk-factors for these. Third, patient-reported outcomes used in this study were subjective by nature which potentially have introduced outcome reporting bias. However, the influence of such bias in our study is considered negligible since participants from both groups were unaware of illness aetiology during study conduct. Finally, data on some potentially important predictors of a complicated course such as respiratory rate and peripheral oxygen saturation were not available for all included patients which precluded inclusion in the prediction analyses.

### Comparison with existing literature

While only 15.6% of SARS-CoV-2 positive patients did not report full recovery at 28 days in our study, previous reports of non-hospitalised COVID-19 patients during the early phases of the pandemic showed significantly higher rates of persistent symptoms including fatigue and shortness of breath; at least one symptom was reported in 53.1% of patients after a mean of 125 days [2], 36% at >4 weeks [3], and 28% at month 4 post-infection [6]. To date, the true incidence of long-COVID remains to be elucidated. Estimates across studies vary widely due to substantial heterogeneity in study population and study methods, and results were often not compared to a proper comparison group, nor controlled for pre-existing issues, symptoms that would have occurred anyway, or selection and reporting/recall bias. The World Health Organisation estimates the percentage of people who continue to have, or develop, a least one symptom more than 3 months after SARS-CoV-2 infection as 10-20% [11]. However, a meta-analysis of 194 studies estimated that, 35% of non-hospitalised COVID-19 survivors went on to experience at least one unresolved symptom at about 4 months [12]. A recent Scottish nationwide population cohort study, taking background rates and confounding into account, found that at least one symptom was reported in 65% of adults 6 months following SARS-CoV-2 infection while this was also reported in 51% of age-, sex-, and socioeconomically-matched and never-infected adults [13]. Following adjustment for potential confounding, the difference in prevalence of one or more symptom attributable to SARS-CoV-2 infection at 6 months dropped (from 14%) to 7%. These observations closely resemble our finding that recovery at 28 days did not substantially differ between SARS-CoV-2 positive and negative patients.

Our study found no found no evidence that candidate predictors of a complicated course, including not yet feeling fully recovered at day 2, differed between patients with RTI symptoms who did and did not tested positive for SARS-CoV-2. The included candidate predictors closely resembled those found to be associated with (a cluster of) symptoms after 6 months of SARS-CoV-2 infection (illness severity, age, BMI, smoking, pre-existing comorbidity and female sex) in a German large population-based study [14]. Similarly, a large US retrospective cohort study found illness severity, age and obesity associated with long-term adverse outcomes [15].

### Implications for research and practice

Albeit the average time to self-reported full recovery was approximately 3 days longer in SARS-CoV-2 positive patients, recovery at 28 days did not substantially differ between SARS-CoV-2 positive and negative patients. Nevertheless, the observed mean difference of 3 days is generally considered clinically meaningful. Any between-group differences observed in hospital admission and complicated course should be interpreted with great caution since these were derived from unadjusted analyses. Also, we did not aim to assess such differences with our study but rather explore whether predictors of a complicated course differed between patients with RTI symptoms who tested either SARS-CoV-2 positive of negative. In our study, rhinovirus was the most common viral pathogen detected in SARS-CoV-2 negative patients while influenza and Respiratory Syncytial Virus (RSV) were not detected [10]. Since influenza and RSV are associated with more severe course of illness than rhinovirus, we anticipate that any observed differences between groups in our study are likely to be smaller in seasons where influenza and RSV are more prevalent.

Full recovery at 28 days was reported in 84% of SARS-CoV-2 positive patients and 82% of SARS-CoV-2 negative patients. This shows that persisting symptoms following infection is not a SARS-CoV-2 specific phenomenon which is in line with previous reports of other infectious viral and non-viral diseases [7,8]. Increasing public awareness about this phenomenon is highly important.

The findings of our study demonstrate the need of research with appropriate comparison groups early in a pandemic to mitigate the risk of bias due to selective outcome reporting influenced by its sudden high prevalence, media attention and public perceptions about the illness. Future research is needed to unravel which host- and pathogen-related profiles are associated with the occurrence of complications and persisting symptoms among patients presenting in primary care with a RTI.

## Conclusion

Among patients presenting in primary care with RTI symptoms early in the COVID-19 pandemic, 28 days recovery was comparable between SARS-CoV-2 positive and negative patients, but it took somewhat longer for SARS-CoV-2 positive patients to feel fully recovered. We found no evidence that that predictors of a complicated course differed between SARS-CoV-2 positive and negative groups.

## Data Availability

Data are available upon request, explaining research question and methods, from the last author (AWV) who will seek agreement from the core research team.

## Public and patient involvement

Patients were not involved in the design or conduct of the study.

## Trial registry

The study was registered in the Dutch Trial Register (NL8520; https://www.onderzoekmetmensen.nl/nl/trial/29166)
